# The role of methylprednisolone in severe COVID-19 patients: a meta-analysis

**DOI:** 10.3389/fmed.2024.1428581

**Published:** 2024-08-09

**Authors:** Wanru Xu, Yujun Zeng, Hedong Han, Tangfeng Lv, Dang Lin

**Affiliations:** ^1^Department of Respiratory and Critical Care Medicine, The Affiliated Suzhou Hospital of Nanjing Medical University, Suzhou Municipal Hospital, Nanjing Medical University, Suzhou, China; ^2^Department of Respiratory and Critical Care Medicine, Jinling Hospital, School of Medicine, Nanjing University, Nanjing, China

**Keywords:** COVID-19, severe, methylprednisolone, mortality, adverse event

## Abstract

**Background:**

The purpose of this study is to assess the effectiveness of methylprednisolone in severe COVID-19.

**Methods:**

PubMed, the Cochrane Library and Web of Science were searched for literatures comparing methylprednisolone and control treatment in severe COVID-19 patients. Statistical pooling was reported as risk ratio (RR) with corresponding 95% confidence interval (CI). The outcomes of interest in the literature survey were mortality and adverse events.

**Results:**

A total of 13 studies were included, including 3,138 patients with severe COVID-19, of which 1,634 patients were treated with methylprednisolone and 1,504 patients were treated with control treatment. Five of the 13 studies reported severe adverse events. Our meta-analysis indicates that methylprednisolone treatment in COVID-19 patients is associated with a significant reduction in mortality (RR 0.62, 95% CI 0.46–0.85, *p* = 0.003) compared to control treatment, without an increased risk of adverse events (RR 1.20, 95% CI 0.92–1.56, *p* = 0.17). Moreover, high-dose methylprednisolone treatment (RR 0.57; 95% CI 0.40–0.82, *p* = 0.003) and short-course methylprednisolone treatment (RR 0.54; 95% CI 0.38–0.89, *p* = 0.01) found to significantly reduce mortality. Additionally, it was found that younger severe COVID-19 patients (RR 0.40; 95% CI 0.20–0.80, *p* = 0.01) had better outcomes to methylprednisolone than older patients.

**Conclusion:**

Methylprednisolone was correlated with lower mortality compared with control treatment in severe COVID-19 patients without increasing serious adverse reactions. Furthermore, high-doses and short-term of methylprednisolone treatment were linked with better younger COVID-19 reported higher benefit from methylprednisolone than older COVID-19 patients.

## 1 Introduction

COVID-19 epidemic rapidly spread worldwide, leading to huge economic, social and health losses. COVID-19, which is a variant of the coronavirus, can result in a range of infection outcomes from asymptomatic carriers to quickly progressing life-threatening disease (such as ARDS) with a high mortality rate (1), primarily affecting older adults with chronic underlying conditions (2). Glucocorticoids have been widely used in treating severe COVID-19, but there are conflicting researches about their effectiveness in treating SARS-CoV-2 infection. Some studies suggest that glucocorticoids should not be used regularly to treat COVID-19 (3). However, the RECOVERY trial reported that dexamethasone reduced the 28-day mortality in COVID-19 patients (4), providing evidence in support of systemic corticosteroid use. Nevertheless, recent studies suggest that methylprednisolone may have a better curative effect in severe COVID-19 patients (5). However, there are still no clear guidelines on the proper dosage, duration, and treatment period of methylprednisolone. Therefore, the purpose of this study was to explore the efficacy of methylprednisolone in treating COVID-19, followed by a meta-analysis of relevant literature.

## 2 Methods

### 2.1 Literature search

According to the Cochrane scheme, two authors searched all published articles in PubMed, the Cochrane Library and Web of Science databases by combining subject headings with free words from December 2019 to March 10, 2023. The search terms were “glucocorticoid” or “corticosteroid” or “Methylprednisolone” AND “COVID-19” or “SARS-COV-2.” Additionally, we screened the references of correlated articles.

### 2.2 Inclusion and exclusion criteria

The inclusion criteria were as follows: (1) Subjects: Patients who met the diagnostic criteria for severe or critical COVID-19 (2). (2) Type of study: randomized controlled study, cohort study and case–control study. (3) Intervention measures: the Methylprednisolone group received conventional treatment along with methylprednisolone, the control group was only used with conventional treatment. There were no restrictions on the dose or administration method of methylprednisolone for inclusion in the study. (4) Study results: the primary outcome was mortality, and the secondary outcome was adverse event incidence.

The exclusion criteria were as follows: (1) patients who did not have severe COVID-19 or were not treated with methylprednisolone. (2) Literature review, systematic review, meta-analysis, case reports, animal tests, guidelines, etc. (3) Literature with repeated publication, incomplete data and unrigorous experiments. (4) Mortality was not reported in the Results section. (5) Control group was not established in studies.

### 2.3 Data extraction

Two researchers independently reviewed the titles and abstracts of literatures to identify possible related studies. After a thorough text review, relevant literature was included and data was collected. Any disputes between the two researchers were resolved with the assistance of the third researcher. The data extracted included baseline information such as author, publication year, study interval, sample size, and population characteristics. Additionally, intervention measures including methylprednisolone prescription were recorded, along with study endpoint and results measurement data.

### 2.4 Risk assessment

The Risk of Bias tool suggested by the Cochrane Handbook 5.1.0 was utilized to assess the bias risk for the included RCTs. The Newcastle-Ottawa Scale (NOS) was used to assess the quality of the included cohort and case–control studies, which consists of 3 main aspects: study selection (0–4 points), comparability (0–2 points), and exposure factors or outcomes (0–4 points). A total score of 8 or 9 was assessed as good quality, 6 or 7 as fair quality, and ≤5 as poor quality. The above steps were independently evaluated and cross-checked by 2 investigators, and if discussions failed to reach a consensus, they were discussed by both parties or resolved after conferring with a third investigator.

### 2.5 Statistical analysis

Review Manager 5.4.1 was utilized for the statistical analysis. Risk ratios (RR) and their associated 95% confidence intervals (CI) were used to evaluate dichotomous data. Heterogeneity was determined quantitatively by *X*^2^ test and *I*^2^. If *p* > 0.1 and *I*^2^ < 50%, no heterogeneity was considered and fixed effects model was adopted. If *p* < 0.1 or *I*^2^ > 50%, significant heterogeneity was suspected, thus implementing a random effects model for analysis. Besides, a sensitivity analysis and subgroup analysis were conducted to further examine the factors of the heterogeneity. Additionally, Stata 15.0 was utilized to carry out the Egger test, Begg test, cut-and-patch method, and funnel plot that collectively evaluated publication bias. *p* < 0.05 was considered statistically significant.

## 3 Results

### 3.1 Search results

The flow chart of the literature search is shown in Supplementary Figure S1. Our study consisted of a total of 13 eligible studies (1, 6–17), consisting of 10 observational studies and three randomized controlled trials. Three thousand one hundred thirty-eight patients with severe COVID-19 were reviewed, of whom 1,634 in the control group and 1,504 in the methylprednisolone group. All studies reported mortality data, while five studies reported serious adverse events. The baseline characteristics of the included studies are displayed in Supplementary Table S1.

### 3.2 Quality evaluation of research literature

The risk assessment methods recommended by the Cochrane Collaboration were utilized to assess the quality of RCTs (Supplementary Figure S2). Meanwhile, the observational studies were rated using the NOS, with scores ranging from 6 to 8 points, indicating an overall good quality (Supplementary Table S2).

### 3.3 Meta-analysis

#### 3.3.1 Main results

**Figure 1:**
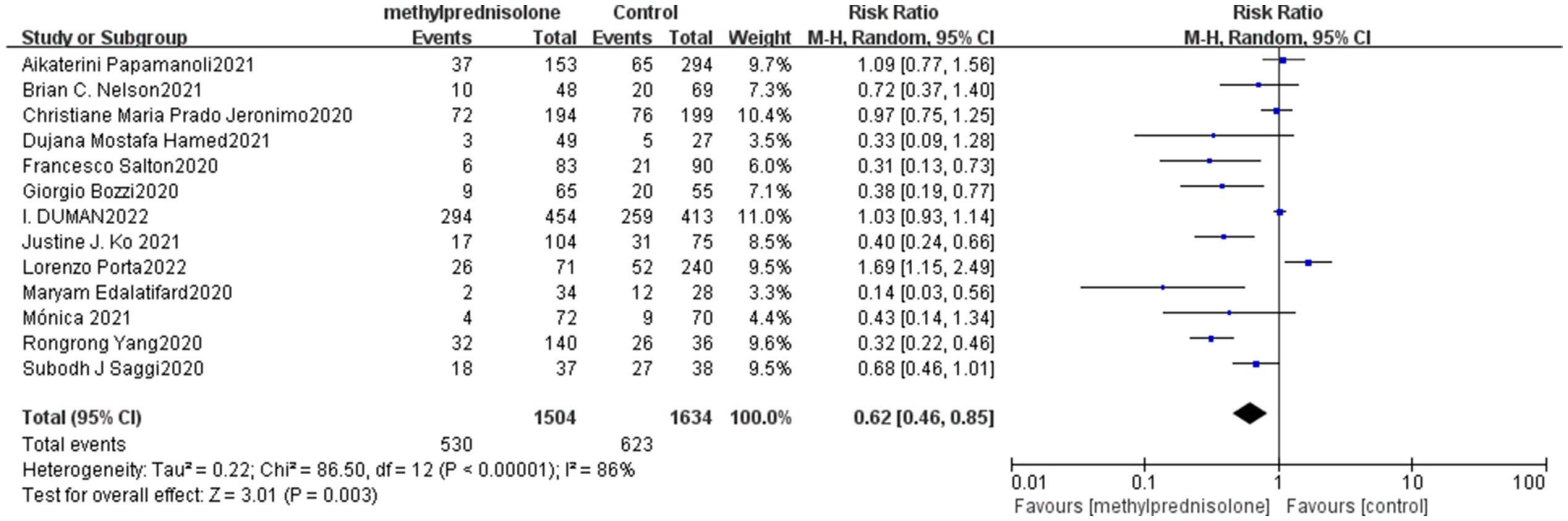
Forest plot of the mortality among patients with serve COVID-19 in Methylprednisolone group vs. Control group.

In all 13 studies reviewed, among the 3,138 severe COVID-19 patients evaluated, 530 (35.2%) of 1,504 severe patients who received methylprednisolone died, compared to 623 (38.1%) of 1,634 patients who did not receive methylprednisolone. The result of the meta-analysis suggest that the use of methylprednisolone significantly reduced the death rate of patients with severe COVID-19 (RR 0.62, 95%CI 0.46–0.85, *p* < 0.01), as shown in Figure 1.

#### 3.3.2 Secondary results

**Figure 2:**
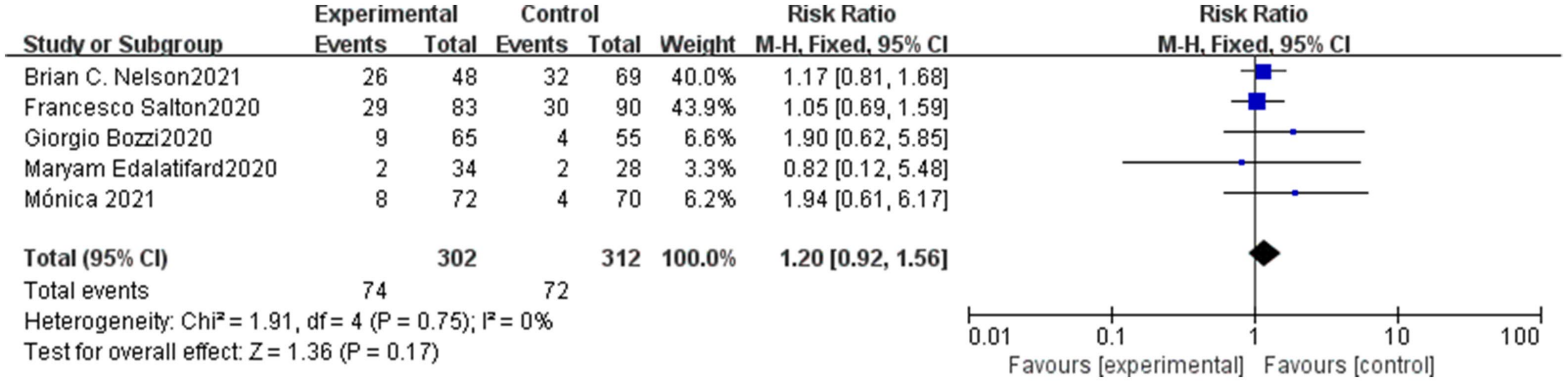
Forest plot of the serve advent events rate of serve COVID-19 patients in Methylprednisolone group vs. Control group.

Our meta-analysis identified five studies that reported data on severe adverse reactions following treatment in both the methylprednisolone and control groups. Serious adverse reactions occurred at a rate of 24.5% (74/302) in the methylprednisolone group and 23.1% (72/312) in the control group. The most commonly reported severe adverse reactions were sepsis and gastrointestinal bleeding. However, there was not a significant distinction between the methylprednisolone group and the control group in the probability of developing severe adverse events (RR 1.20, 95%CI 0.92–1.56, *p* = 0.17), as shown in Figure 2.

#### 3.3.3 Subgroup analysis

**Figure 3:**
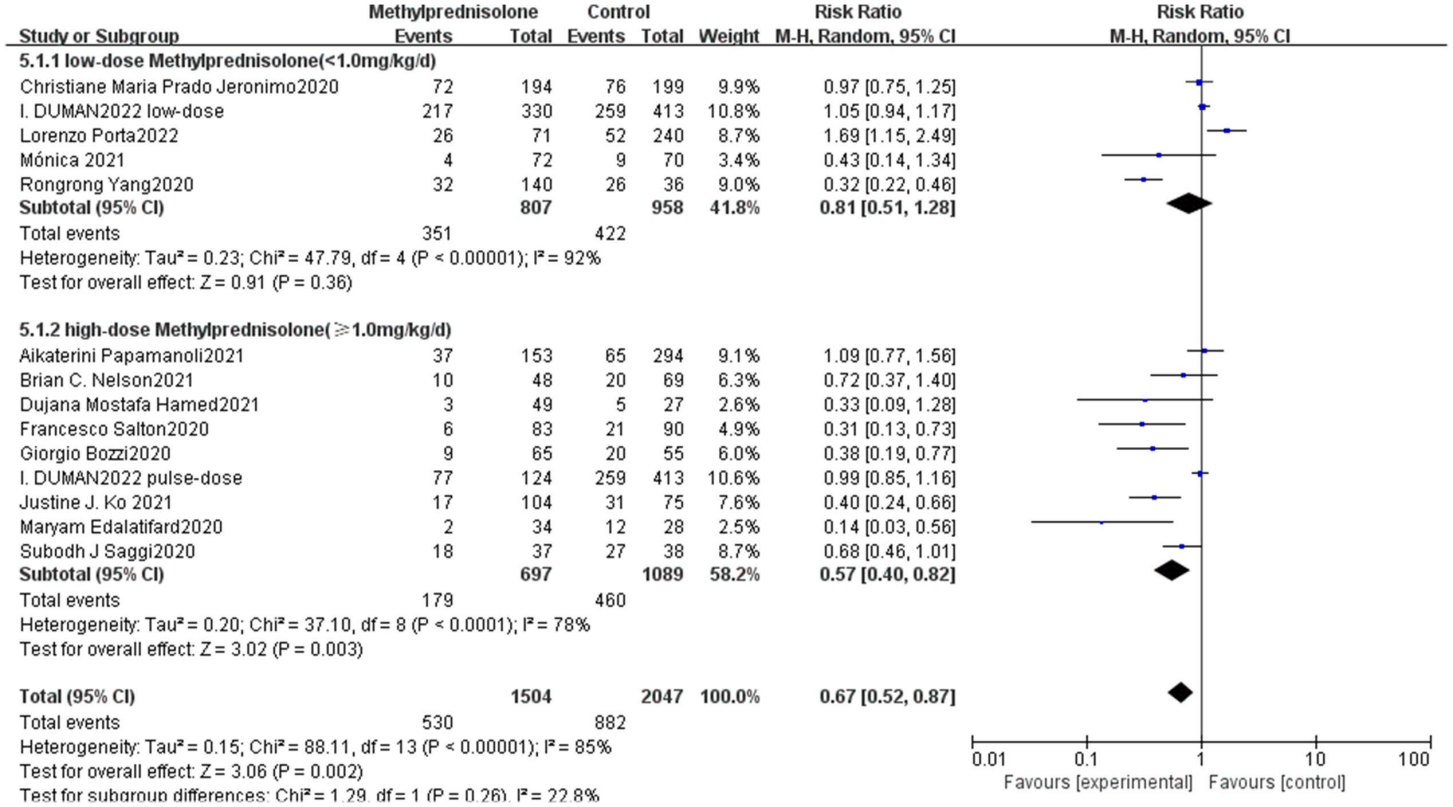
Forest plot of the association between the different doses of methylprednisolone and mortality rate in patients with serve COVID-19.

In this study, we analyzed different doses of methylprednisolone according to the dosage used. In the study of I. DUMAN, the methylprednisolone group has been divided into a low-dose group and a pulse group. Therefore, nine studies involved high-dose methylprednisolone (methylprednisolone ≥ 1.0 mg/kg/d) and five studies involved low-dose methylprednisolone (methylprednisolone < 1.0 mg/kg/d). Notably, high-dose methylprednisolone was significantly associated with reduced mortality of severe COVID-19 patients (RR 0.57; 95% CI 0.40–0.82, *p* = 0.003), as shown in Figure 3. However, patients with severe COVID-19 mortality did not significantly decrease in the low-dose methylprednisolone group (RR 0.81; 95% CI 0.51–1.28, *p* = 0.36).

**Figure 4:**
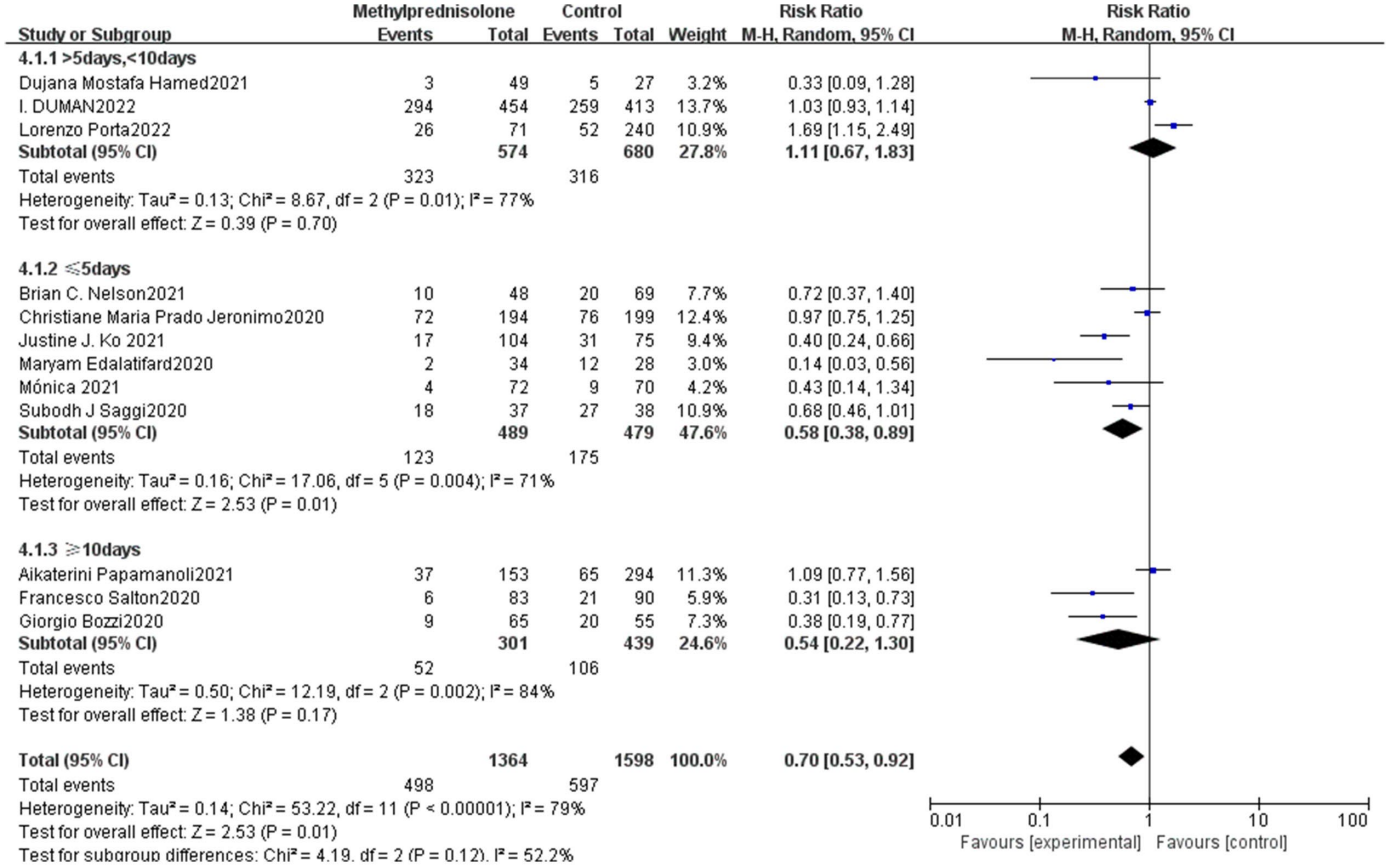
Forest plot of the mortality among patients with severe COVID-19 in different time of methylprednisolone administration.

The study included subgroup analyses based on the methylprednisolone treatment course among which one of the studies did not report this information for severe COVID-19 patients, and the remaining 12 studies were divided into long course, medium course and short course according to the treatment time of methylprednisolone. Three studies were classified as long-term treatment (≥10 days), six studies as short-term treatment (≤5 days), and three studies as medium-term treatment (>5 days, <10 days). The results showed that the long course of methylprednisolone, (RR 0.54; 95% CI 0.22–1.30, *p* = 0.17) moderate treatment (RR 1.11; 95% CI 0.67–1.83, *p* = 0.70) had no significant correlation with the mortality of severe patients. Whereas, short course of methylprednisolone significantly reduced the mortality of patients with severe COVID-19 (RR 0.54; 95% CI 0.38–0.89, *p* = 0.01). The result is shown in Figure 4.

**Figure 5:**
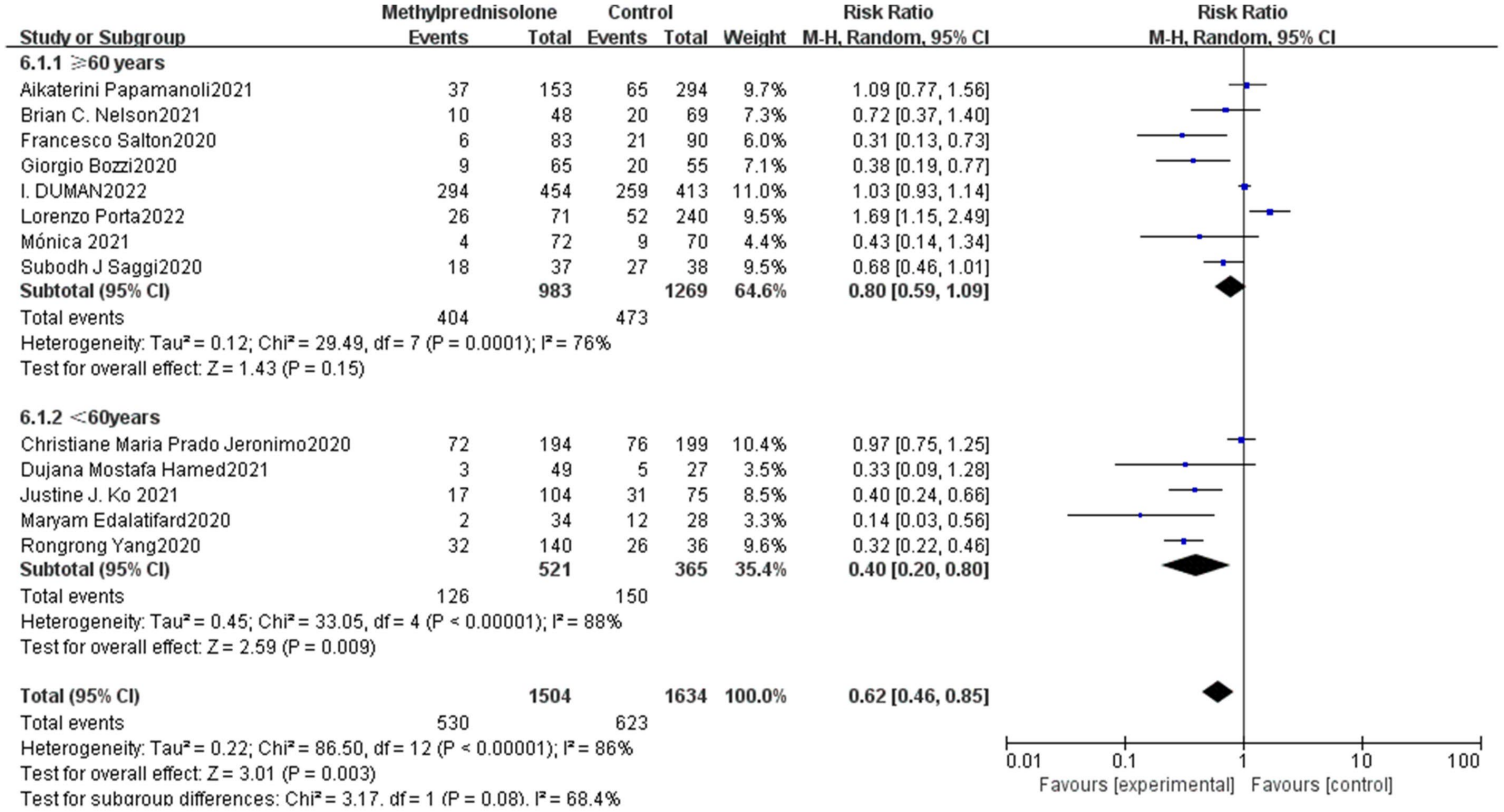
Forest plot of the mortality among patients with severe COVID-19 in different age.

Subgroup analysis was carried out in terms of the median age of patients. Eight studies were elderly (≥60 years) and five studies were young adults (<60 years). The results showed that young patients (<60 years) had a significantly lower mortality (RR 0.40; 95% CI 0.20–0.80, *p* = 0.01), whereas the elderly group (≥60 years) did not show a notable connection with reduced mortality (RR 0.81; 95% CI 0.99–1.09, *p* = 0.15). The result is shown in Figure 5.

Besides, we performed subgroup analyses by study type, geographic locations and sample size (Supplementary Table S3). The analyses revealed that in observational studies, methylprednisolone reduced mortality among patients with severe COVID-19. Furthermore, the results indicated there were no notable differences in study findings based on geographic location or sample size.

### 3.4 Sensitivity analysis and publication bias

The ‘leave-one-out’ method was used to conduct the sensitivity analysis of the study. Sensitivity analysis showed that the aggregate effect size remained unaffected when each study was in turn removed, suggesting that the results were stable.

The funnel plot of the study (see Supplementary Figure S3) showed poor symmetry, as evidenced by the Egger’s test *p*-value of 0.02. This suggests the potential for publication bias in reporting results. However, the *p*-value of Begg’s test was 0.13, and the effect values were adjusted using the cut-and-patch method. The results demonstrated that they were consistent with the pre-correction values, supporting the stability of the results.

## 4 Discussion

We analyzed data from 13 studies comprising 3,138 patients to evaluate the effectiveness of methylprednisolone in severe COVID-19 patients. We also analyzed data from 5 studies assessing the risk of severe adverse reactions associated with methylprednisolone treatment for COVID-19 patients. Our findings suggest that methylprednisolone administration in treating severe COVID-19 patients is advantageous, leading to reduced mortality rates without increasing the risk of serious adverse reactions when compared to the treatment without glucocorticoids.

The COVID-19 pandemic has swept the globe, seriously affecting human health and life. Studies suggested that the treatment of corticosteroids could reduce mortality rates among severe COVID-19 patients. However, the results have been inconsistent. Research indicates that after infecting host cells, SARS-CoV-2 causes endothelial and alveolar cell inflammation and injury by inducing immunological and inflammatory responses (18, 19). Particularly, Severe COVID-19 patients have depleted antiviral defense systems, leading to life-threatening systemic inflammatory responses (15). Then viral ‘sepsis’ may occur with severe complications, including inflammatory lung injury, acute respiratory distress syndrome (ARDS), different organ failures, and ultimately death (18, 20). Corticosteroid as a classic immunosuppressive drug could delay or prevent pneumonia progression and improve ARDS (21). At the same time, corticosteroid as an anti-inflammatory agent could reduce serum levels of proinflammatory mediators, suppress lung and systemic inflammatory responses, and rapidly improve lung damage, thereby reducing mortality rates (18). Nevertheless, some studies indicate that glucocorticoid may fail to reduce mortality rates and would result in serious adverse reactions, such as secondary infections that could worsen the disease. The RECOVERY trial demonstrated glucocorticoid advantages in severe COVID-19 treatment (4). However, this study used dexamethasone, which has been reported that has lower *in vitro* responses than methylprednisolone, the preferred type of glucocorticoid for treating lung diseases like acute rejection of lung transplantation and pneumonia caused by pneumonia (10, 22). A study by Ko et al. (10) comparing methylprednisolone and dexamethasone found that methylprednisolone was superior to than dexamethasone in reducing mortality rates among patients with severe COVID-19. Severe COVID-19 patients may experience rapidly developing lung injury, leading to high mortality rates. Methylprednisolone could have higher lung penetration than dexamethasone, contributing to its better effectiveness among severe COVID-19 patients.

The prospective study by Lorenzo Porta et al. demonstrated that routine use of methylprednisolone could not be effective in lowering short-term mortality in patients, specifically those with cardiovascular or respiratory complications, and may raise the likelihood of shock or acute respiratory failure (13). Conversely, the retrospective study found that intravenous methylprednisolone effectively reduced mortality among COVID-19-induced ARDS patients, while also improving the likelihood of recovering renal and lung function (14). In a meta-analysis similar to our own, methylprednisolone decreased short-term mortality, the requirement for mechanical ventilation, and ICU admission, but increased the viral clearance time in COVID-19 patients (22). However this analysis encompassed patients with mild, moderate, severe, and critical illness. Our meta-analysis also supports that methylprednisolone could be used to treat severe COVID-19 patients, since it significantly reduces mortality without increasing the occurrence of serious adverse reactions.

Currently, several studies have showed methylprednisolone could be an effective treatment for severe COVID-19 and may help reduce mortality rates. However, there is currently a lack of clear guidelines regarding the most appropriate therapeutic dosage, course, and duration of methylprednisolone. To address these issues, we performed subgroup analysis of the methylprednisolone dosage and course, examining the effects of varying quantities of the drug on severe COVID-19 patients. The result of subgroup suggested that doses greater than 1.0 mg/kg/d of methylprednisolone were associated with reduced mortality among severe patients. Notably, the retrospective study by Papamanoli et al. (12) also found that high-dose methylprednisolone treatment lowered mortality rate and mechanical ventilation among severe patients. Previous research has demonstrated that the treatment of lung diseases often requires a direct effect of high-dose glucocorticoids on cell membrane-related proteins, which could potentially offer greater efficacy than lower doses of methylprednisolone (10, 23). However, some studies do not advocate the use of high-dose glucocorticoids, possibly considering that high-dose glucocorticoids are more likely to cause serious adverse effects (24). In subgroup analysis based on the duration of methylprednisolone, the use of short-term glucocorticoids (≤5 days) showed a significant reduction in mortality. Short-term use of glucocorticoids (3–5 days) was also suggested by Parasher (25) for COVID-19 patients who deteriorate based on oxygenation index, rapid imaging progress, and excessive inflammatory response. In contrast, Salton’s study found that early administration of low-dose (80 mg/d) methylprednisolone for a duration longer than 7 days was related with lower mortality and reduced dependence on ventilator (15). Our study had limited literature, only a few studies included for 5–10 days or ≥10 days and this may have produced biased outcomes. Further prospective studies are required to precisely compare the timing of methylprednisolone use. Our subgroup analysis based on patient age showed that methylprednisolone produced better outcomes in severe COVID-19 patients aged below 60 years old. This may be due to the frequent occurrence of underlying diseases and complications among elderly patients, leading to multiple confounding factors that could affect patient prognosis. Although the optimal timing of hormone administration remains controversial, the lack of available literature in our analysis prevented a detailed assessment of methylprednisolone initiation time. Thus, more rigorous studies are needed to clarify the indications, optimal initiation time, and appropriate duration of glucocorticoid therapy.

Our meta-analysis also has some limitations. First, our study consisted of mostly retrospective studies and further prospective studies are still urgently needed. Secondly, owing to the high heterogeneity of data analysis, the possibility of migration is high. To mitigate this concern, sensitivity analysis and subgroup analysis were performed to minimize the impact of such variability. Thirdly, the definition and reporting of serious adverse events vary between trials. Furthermore, only a few of the included studies provided records of serious adverse events.

In conclusion, in severe COVID-19 patients, our analysis shows that the use of methylprednisolone is associated with reduced mortality when compared to conventional treatment. Furthermore, our analysis confirmed that high doses and short courses of methylprednisolone were significantly associated with increased mortality. In the clinic, Short-term high dose methylprednisolone treatment could be administered to the severe COVID-19 patients.

## Data availability statement

The original contributions presented in the study are included in the article/Supplementary material, further inquiries can be directed to the corresponding authors.

## Author contributions

WX: Investigation, Methodology, Software, Writing – original draft. YZ: Data curation, Methodology, Writing – original draft. HH: Conceptualization, Data curation, Methodology, Software, Validation, Writing – original draft. TL: Supervision, Validation, Writing – review & editing. DL: Writing – review & editing, Supervision, Validation.
