# Supplementary material for: The role of methylprednisolone in severe COVID-19 patients: a meta-analysis
Source: Front Med (Lausanne). 2024 Aug 9;11:1428581. doi: 10.3389/fmed.2024.1428581 (PMC11341412; doi:10.3389/fmed.2024.1428581)
Supplement: Supplementary file 1 [file Data_Sheet_1.pdf]

Supplementary Material

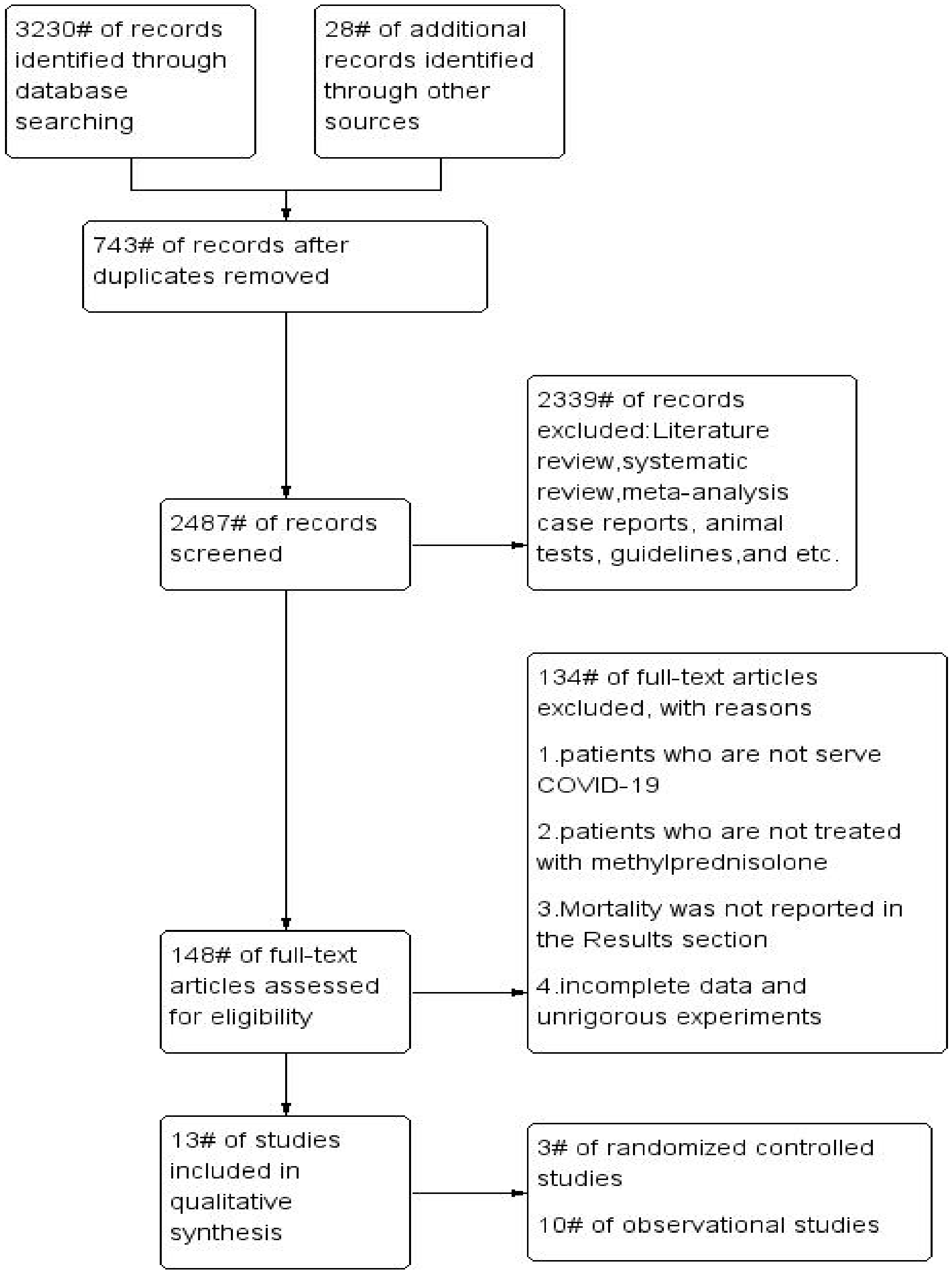

Fig. 1: Flow diagram of including of relevant studies

* These authors contributed equally to this manuscript.

** These authors contributed equally to this manuscript.

Corresponding author: Dang Lin, Baita West Road 16,215000 Suzhou, China. Email: lind69@163.com

Alternate corresponding author: Tangfeng Lv, MD. East Zhongshan Road 305, 211100 Nanjing, China. Email: bairoushui@163.com.

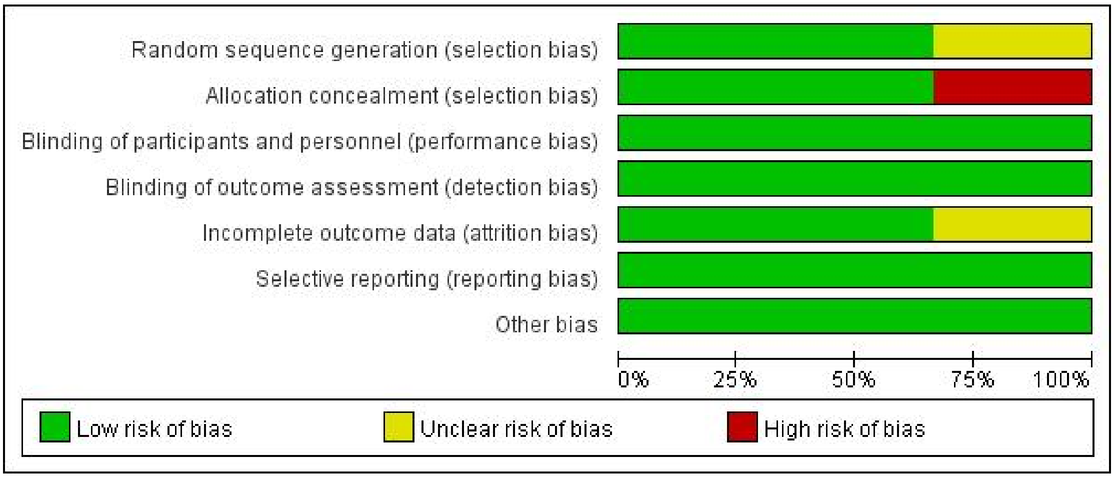

Fig. 2: Quality assessment of included RCT studies

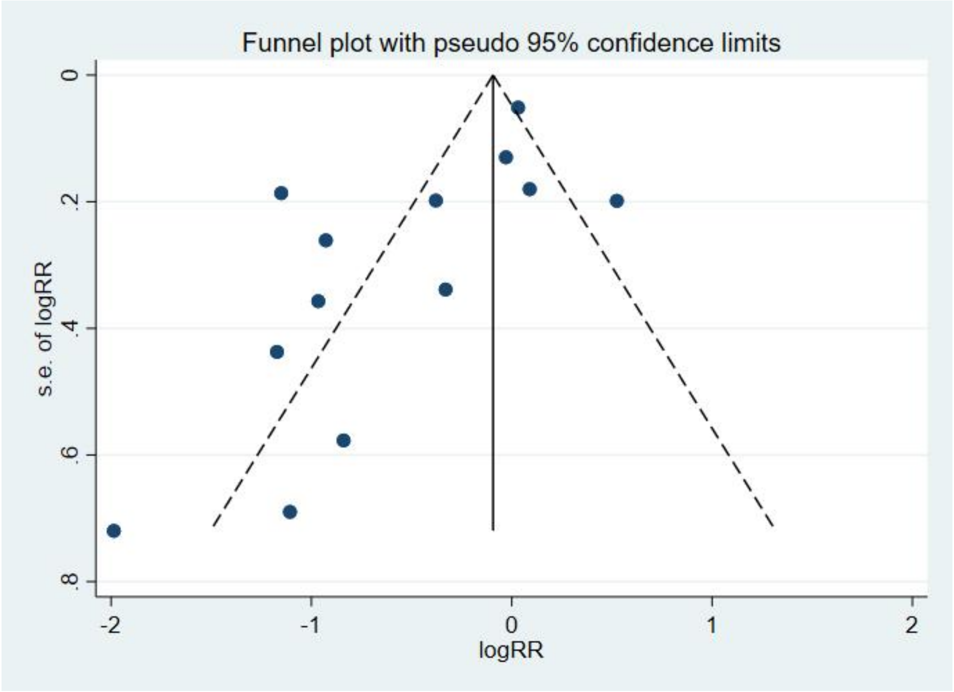

Fig 3**.** Funnel plots of included studies

Table 1 The characteristics of the included studies.

Author	Year	Area	Interval	Total	Mean age	Male proportion	Study design	Methylprednisolone Prescription	Outcome
Aikaterini Papamanoli	2021	America	March 1,2020 to April 15, 2020	447	61.3	65.1%	Retrospective cohort study	Median dose, 1.78 mg/kg/ day; Median duration,10days	28-day mortality, mechanical ventilation
Brian C. Nelson	2021	America	March 1 ,2020 to April 12, 2020	117	63	68.3%	Case-control study	1.0 mg/kg/day for 5 days	28-day without ventilators, mortality, hospital discharge,
Dujana Mostafa Hamed	2021	Dubai	April 20, 2020 to June 20, 2020	76	48	85.5%	Randomized controlled study	80mg/day for 7 days	mortality, ICU admissions, length of ICU stays, days on ventilator, and length of stay
Francesco Salton	2020	Dubai	February 27,2020 to April 24, 2020	173	64.6	69.4%	Retrospective cohort study	80 mg/day for at least 8 days,	ICU referral, intubation, or 28-day mortality
Giorgio Bozzi	2020	Italy	February 25,2020 to March 30, 2020	120	62	80.0%	Prospective cohort study	1 mg/kg/day for 5 days, then 0.5 mg/kg/day for 5 days, and then 0.25 mg/kg/day until day 14	mortality, ICU admission, length of ICU stays, days on ventilators, length of hospital stay

I. DUMAN	2022	Turkey	July 1, 2020 to February 28, 2021	867	66.38	60.4%	Retrospective cohort study	0.5 to 1 mg/kg/day for 5 to 10 days	28-day mortality
Lorenzo Porta	2022	Italy	March 1,2020 to April 30, 2020	311	63.1	64.6%	Retrospective cohort study	0.5-1mg/kg/day for 4-7 days	30-day mortality
Maryam Edalatifard	2020	Turkey	April 20, 2020 to June 20, 2020	62	58.5	62.9%	Randomized controlled study	250 mg/day for 3 days	Time of clinical improvement and discharge from the hospital or death
Rongrong Yang	2020	China	January 1,2020 to March 7, 2020	175	57	60.6%	Retrospective cohort study	50-80 mg/day	NA
Christiane Maria Prado Jeronimo	2020	Brazil	April 18,2020 to June 16, 2020	393	55	64.6%	Randomized controlled study	0.5 mg/kg for 5 days	28-day mortality
Monica Climente- Marti	2021	Turkey	March 9, 2020 to July 2, 2020	142	67.6	55.6%	Retrospective cohort study	0.5 to 1.0 mg/kg/day for less than 5 days	28-day mortality
Subodh J Saggi	2020	America	March 1 ,2020 to April 30, 2020	118	72.74	58.7%	Retrospective cohort study	1–2 mg/kg/day for 5–7 days	28-day mortality
Justine J. Ko	2021	China	January 20,2020 to February 25, 2020	179	56.3	76.0%	Retrospective cohort study	1 mg/kg/day for 3 days	50-day mortality

Table 2 Quality assessment of observational study using Newcastle Ottawa scale.

Author	year	Selection	Comparability	Exposure	Total Score	Quality of the Study
Aikaterini Papamanoli	2021	3	1	3	7	fair
Brian C. Nelson	2021	3	2	3	8	good
Francesco Salton	2020	3	1	2	6	fair
Giorgio Bozzi	2020	3	2	3	8	good
I. DUMAN	2022	2	1	3	6	fair
Lorenzo Porta	2022	2	2	2	6	fair
Rongrong Yang	2020	4	1	2	7	fair
Mónica Climente-Martí	2021	3	1	2	6	fair
Subodh J Saggi	2020	2	1	3	6	fair
Justine J. Ko	2021	2	1	3	6	fair

Table 3. subgroup analysis (study type, geographic locations, sample size) of methylprednisolone for severe COVID-19 patients

subgroup		Study (No.)	I^2 (%)	P (I^2)	RR	P(RR)
Study type	Observational study	10	88	<0.001	0.64(0.44-0.92)	0.02
	RCT	3	79	0.008	0.41(0.11-1.48)	0.17
Geographic locations	Asia	4	87	<0.001	0.83(0.50-1.39)	0.48
	Europe	5	78	0.001	0.49(0.24-0.97)	0.04
	America	4	89	<0.001	0.49(0.22-1.11)	0.09
Sample size	>300	4	81	0.001	0.70(0.42-1.15)	0.16
	≤300	9	85	<0.001	0.59(0.38-0.91)	0.01
